# Benchmarking mobilization practice and functional outcomes in traumatic brain injury patients admitted to the intensive care unit: a three-year service evaluation

**DOI:** 10.3389/fneur.2026.1694393

**Published:** 2026-01-26

**Authors:** Fiona Howroyd, James Hodson, Anne Preece, Tammy Lea, Samantha Rooney, Hon Sing Geoffrey Wu, Simran Rahania, Fang Gao Smith, Tonny Veenith, Niharika A. Duggal, Zubair Ahmed, Jonathan Weblin

**Affiliations:** 1 Queen Elizabeth Hospital Birmingham, University Hospitals Birmingham NHS Foundation Trust, Birmingham, United Kingdom; 2 Department of Inflammation and Ageing, School of Infection, Inflammation and Immunology, College of Medical and Health, University of Birmingham, Birmingham, United Kingdom; 3 School of Sport, Exercise and Rehabilitation, University of Birmingham, Birmingham, United Kingdom; 4 Royal Wolverhampton Hospital, Wolverhampton, United Kingdom; 5 Institute of Acute Care, University of Wolverhampton and Royal Wolverhampton Hospital, Wolverhampton, United Kingdom

**Keywords:** critical care, head injury, major trauma, mobilization, neurotrauma, rehabilitation

## Abstract

**Background:**

Traumatic brain injury (TBI) is a major cause of death and disability worldwide. Mobilization is defined as the application of assisted movement and physical therapy to hospitalized patients, including progressive exercise and ambulation programs. While early mobilization in the intensive care unit (ICU) has been shown to be a safe and effective intervention to improve patient outcomes in the general ICU cohort, there is currently limited evidence specific to patients with acute TBI. The aim of this service evaluation was to identify current mobilization activity and functional outcomes in patients admitted to the ICU at our institution following an acute TBI.

**Methods:**

A single-center retrospective service evaluation was performed for all patients, over 16 years-old, admitted to the ICU at our institution (a Level 1 major trauma center) with an acute TBI between January 2022 and November 2024. Patient demographics, ICU admission details, TBI severity (based on the Glasgow Coma Scale [GCS]) and functional outcomes were extracted. Mobilization outcomes included the timing of the commencement of mobilization (defined as sitting on the edge of the bed or better) and mobilization status, defined using the Manchester Mobility Scale (MMS).

**Results:**

The service evaluation included 353 patients, of whom 56.0% had severe TBI (GCS: 3–7). Mobilization was achieved in ICU for 53.0% of patients, with a further 18.1% first mobilized on a hospital ward post-ICU discharge. The first mobilization occurred at a median of 11 days (interquartile range: 6–18) after ICU admission. In patients surviving to ICU discharge, 28.9% had an MMS of 1 (bed-based exercises) at this time, with only 9.1% achieving an MMS of 7 (mobilizing 30 meters or more). Analysis by TBI severity found a significant decline in in-hospital mobilization rates with increasing TBI severity (90.7% vs. 58.4% for mild vs. severe TBI; *p* < 0.001), with a corresponding increase in the time to the first mobilization (median: 6 vs. 13 days for mild vs. severe TBI; *p* < 0.001).

**Conclusion:**

Acute TBI patients admitted to the ICU at our institution had low rates of mobilization and achieved low levels of mobility at ICU discharge. This service evaluation highlights the need for prospective studies into early mobilization practices in the neurotrauma ICU.

## Graphical abstract

**Figure:**
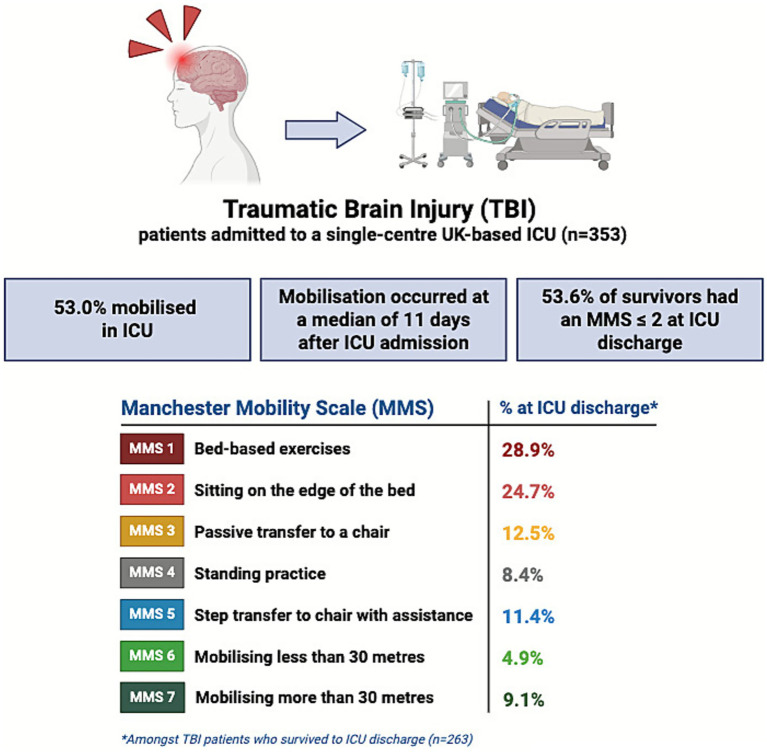
Created in BioRender. Sardeli, A. (2026) https://BioRender.com/w84u9jj.

## 1 Introduction

Traumatic brain injury (TBI) is a major cause of death and disability in all ages, with an annual incidence rate of 69 million worldwide (1). TBIs account for approximately 37% of all injury-related deaths across Europe (2). However, global mortality rates vary, with lower mortality observed in countries with higher levels of human development, as measured by life expectancy, education and income measures (3, 4). Lower mortality rates are also observed in specialist neurotrauma intensive care units (ICU), compared to medical-surgical ICU care (5). Survivors of TBI may be affected by long-term physical disability, cognitive disorders and psychological disturbances, affecting functional independence and quality of life (6, 7). As one of the most common causes of death and disability in adults aged under 45 years, TBIs have profound societal impacts and, thus, require urgent attention to improve patient outcomes (6).

Early mobilization in the ICU is a safe and effective intervention to improve patient outcomes after critical illness and is recommended in national guidelines (8, 9). Mobilization is defined as “the application of assisted movement and physical therapy to hospitalized patients,” and is generally achieved using progressive exercise programs, ranging from passive range of movement, to sitting, standing and walking (10). Previous studies have found numerous benefits of ICU mobilization, including reduced durations of mechanical ventilation and ICU stay, as well as improved muscle strength and functional outcomes (11, 12). However, previous early mobilization trials, including those from our own institution, have often focused upon the general ICU population, with many studies excluding those with neurological deficit or traumatic injury (13, 14). While national ICU guidelines advocate early rehabilitation and mobilization during recovery from critical illness, guidelines specific to trauma and neurological injury fail to mention rehabilitation in the ICU (8, 15–18).

The unique complexities of the neurotrauma patient, including altered states of consciousness, cognitive impairment, extra-ventricular drainage devices (EVDs), intracranial pressure monitoring (ICP) bolts and motor and sensory impairments, can make participation and engagement in mobilization activity practically challenging in this cohort (19, 20). However, there are a breadth of rehabilitation interventions and modalities now available, including specialist equipment and technology to enable active and passive participation in early mobilization (21–23). Nonetheless, concerns remain about the safety and physiological effect of postural changes and exercise following acute TBI, particularly the effects upon haemodynamic status in the context of impaired cerebral autoregulation, although evidence is conflicting (19, 24). Alternative evidence suggests that verticalization, including out-of-bed mobilization, can improve arousal, stimulating sensory pathways and postural reactions and enhancing neuroplasticity (10, 25). A number of non-physical benefits have also been associated with increased physical activity, with enhanced angiogenesis, neurogenesis, the release of neurotrophic factors, and neuroplasticity observed in response to exercise (26). Exercise has also been found to increase cerebral blood flow, oxygen extraction and glucose use, and reduce inflammation in adults, which is associated with an improvement in cognition (27). Although numerous benefits of exercise are known, the specific physiological effects of exercise after TBI in the ICU are not yet fully understood.

Recent systematic reviews focusing on mobilization in the trauma ICU have suggested potential benefits of early rehabilitation upon duration of mechanical ventilation, yet very few studies specific to TBI patients have been conducted (20, 28–30). Studies from the neuro-ICU have shown improved functional recovery with early rehabilitation, yet many have evaluated heterogenous cohorts of traumatic and non-traumatic injuries (31–35). Nonetheless, there have been some small studies from across Asia and Europe specific to the TBI cohort, which found that early ICU rehabilitation after TBI is safe and feasible, and is associated with improvements in mobility status, functional outcomes, pneumonia rates and ICU and hospital lengths of stay (36–39). However, there is considerable heterogeneity in the reported treatment protocols, including the timing and type of rehabilitation intervention, as well as variety in outcome measures and study designs (40). Rehabilitation after TBI may consist of a breadth of interventions, including verticalization, tilt-table, spasticity management and sensory programs; yet few studies have explored early mobilization interventions in the ICU. With no known existing studies from the UK, further research is necessary to understand current mobilization practices for TBI patients in the ICU.

### 1.1 Aims and objectives

The primary aim of this service evaluation was to benchmark current mobilization activity and functional outcomes following acute TBI in a UK-based ICU. Secondary aims were to compare mobilization activity and functional outcomes by TBI severity and by the location of first mobilization (i.e., in ICU, or on an inpatient ward post-ICU discharge).

## 2 Methods

### 2.1 Setting

This single-center service evaluation was conducted at the Queen Elizabeth Hospital Birmingham (QEHB). QEHB is a specialist major trauma and neurosurgical center for patients over 16 years of age, and hosts the largest single-site ICU in Europe, with 67 funded Level 3 ICU beds, 27 of which are specifically allocated to trauma and neurosurgery services. Between January 2022 and January 2023, the ICU neurotrauma rehabilitation service comprised four full-time equivalent physiotherapists, resulting in a staffing ratio of one physiotherapist for every 6.75 Level 3 patients, alongside one full-time equivalent occupational therapist. In January 2024, the appointment of a clinical specialist physiotherapist to the neurotrauma service enhanced the staffing-to-patient ratio to 1:5.4. QEHB provides a five-day rehabilitation service (Monday to Friday); during weekends, only emergency respiratory physiotherapy is provided. The nursing team were staffed as per the Guidelines of Intensive Care Provision with 1:1 nursing for Level 3 patients (41). The physiotherapy team has an established early mobilization protocol (42); however, this protocol is not tailored specifically for patients with TBIs, with no standard operating procedures for the mobilization of patients with an EVD or lumbar drains at the time of this service evaluation. As a result, early mobilization practices and decision making were guided by individual clinical judgment, in collaboration with the wider multidisciplinary team. Local venous thromboembolism (VTE) guidelines incorporated impaired mobility and critical illness within the VTE risk assessment framework, and accompanying local patient information advised the maintenance of regular mobility where clinically safe to do so.

### 2.2 Participants

The service evaluation included a convenience sample of all patients admitted to the ICU with an acute TBI between January 2022 and November 2024. The start date of the period was selected since, prior to this, critical care service delivery was impacted due to the direct effects and aftermath of the COVID-19 pandemic (43, 44). Diagnoses of acute TBI were classified as those confirmed by computed tomography (CT) scan in conjunction with a presenting history of traumatic injury. Where patients had multiple ICU stays during the same hospital admission, only the first ICU stay was considered for analysis.

### 2.3 Data collection

Patients meeting the inclusion criteria were identified from a prospectively-maintained department database. Data for the included cases were primarily extracted from the electronic health records system (EHR). Data extraction was performed by two senior ICU physiotherapists who were not directly involved in the care of this patient cohort, in order to minimize bias. Data extraction for the first 20 patients was completed by both physiotherapists concurrently, with the resulting data compared to ensure consistency. Data for subsequent patients were then extracted by one of the physiotherapists. Any queries or ambiguities were discussed by both physiotherapists and resolved on consensus. The variables collected are detailed subsequently.

#### 2.3.1 Cohort characteristics

Patient demographics comprised age and sex. Severity of illness at ICU admission was quantified using the Acute Physiology and Chronic Health Evaluation (APACHE) II scores, which were extracted from Intensive Care National Audit and Research Center (ICNARC) reports; the scores were also converted into estimated probabilities of in-hospital mortality (45, 46). TBI severity was quantified using the Glasgow Coma Scale (GCS) recorded at the scene of the injury, and categorized as severe (GCS: 3–7), moderate (GCS: 8–13) or mild (GCS: 14–15) (16, 47, 48). The GCS at ICU discharge was also recorded for the subgroup of patients who survived to discharge. The presence of other relevant injuries at ICU admission was identified from the EHR, specifically bony injuries to the pelvis, lower limb, chest or spine (with or without spinal cord injury), as listed in imaging reports.

The EHR was then reviewed to identify neurological support including the use of ICU therapies to control and monitor cerebrospinal fluid (CSF), namely EVDs, lumbar drains or ICP bolts. The level of critical care was based on the patient’s care needs, according to the definitions set out by the Intensive Care Society (49). To summarize, Critical Care Minimum Dataset (CCDMS) Level 2 was classified as basic support and monitoring for two or more organ systems, or advanced support and monitoring for one organ (other than mechanical ventilation), while Level 3 support compromised monitoring and support for two or more organ systems at an advanced level, or the need for mechanical ventilation. The number of calendar days on which patients received sedation, invasive ventilation, and neurological support (as defined by CCDMS) were also recorded (50). In patients requiring tracheostomies, the date of placement and removal were recorded.

#### 2.3.2 Mobilization data

At each physiotherapy session, the physiotherapy team assessed a patient’s level of mobility using the Manchester Mobility Scale (MMS) (51). The resulting records were interrogated to identify the date of first mobilization, which was defined as the first session where patients achieved an MMS ≥ 2 (i.e., sitting on the edge of the bed, or better). Where patients were discharged from ICU before achieving this outcome, the EHR was reviewed to identify evidence of the first mobilization on the subsequent hospital wards. Where the MMS was not explicitly stated, this was estimated retrospectively by evaluation of the medical notes that had been documented by the physiotherapy, occupational therapy or nursing teams. The MMS at the first mobilization, the last rehabilitation session prior to ICU discharge, and at hospital discharge were then recorded.

A case note review was additionally performed for the subgroup of patients who were first mobilized post-ICU discharge, to identify the primary reason that mobilization was not achieved in ICU, which were then categorized into common themes. The first 10 sets of case notes were completed concurrently by both reviewers to check for consistency; any queries or ambiguities in subsequent case notes were resolved via consensus.

#### 2.3.3 Outcome data

The development of ICU-acquired pneumonia was identified by reviewing the EHR to identify common key terms (e.g., pneumonia, ventilator-acquired pneumonia [VAP], hospital-acquired pneumonia [HAP]) and reviewing prescribed antibiotics, to identify prescribing patterns consistent with treatment for pneumonia. The total length of stay (LOS) in ICU and in hospital were then calculated based on the dates of admission and discharge. All calculations of durations were performed based on calendar days (e.g., the ICU LOS was defined as the number of calendar days on which a patient was in ICU). Patients who did not receive invasive ventilation or sedation were assigned a value of 0 days for the respective variables. Where patients died in hospital, the date of death was recorded and used to define whether the death was in ICU, or post-ICU discharge. In patients surviving to hospital discharge and who were not transferred to another hospital for ongoing treatment, the discharge destination was also recorded, and classified as either to inpatient rehabilitation or to a patient’s usual place of residence (“home”). The latter was further subdivided based on whether ongoing community rehabilitation services were provided.

### 2.4 Registration

This project was registered locally (CARMS-22399), and deemed to be a service evaluation; as such, ethical approval was not sought. Patients who had opted out of their information being used for research and planning as part of the National Health Service (NHS) National Data Opt-Out were excluded. The results of the service evaluation are reported in accordance with the Strengthening the Reporting of Observational Studies in Epidemiology (STROBE) guidelines (45) (Supplementary File 1).

### 2.5 Statistical methods

Associations between the GCS at ICU admission and ordinal or continuous variables were assessed using Spearman’s correlation coefficients (rho), and quantified using the associated *p*-value. For nominal factors, the GCS at ICU admission was compared between groups using Mann–Whitney U or Kruskal-Wallis tests, for factors with two or more than two levels, respectively. To visualize these associations, averages or rates for each factor were reported within subgroups of GCS corresponding to mild, moderate and severe TBI. Associations with ICU mortality rates were further assessed using a binary logistic regression model, with the GCS at ICU admission as a continuous covariate. The time to mobilization was additionally assessed using a Kaplan–Meier approach, with patients where this was not achieved censored at death or hospital discharge. Comparisons between patients that were first mobilized in the ICU vs. post-ICU were performed using Fisher’s exact tests for nominal variables, and Mann–Whitney U tests for ordinal or continuous variables.

All analyses were performed using IBM SPSS v29 (IBM Corp. Armonk, NY), with *p* < 0.05 deemed to be indicative of statistical significance throughout. Continuous variables were summarized as means ± standard deviations (SDs) where approximately normally distributed, or as medians and interquartile ranges (IQRs) otherwise. Patients with missing data, or for whom a factor was not applicable were excluded from the analysis of the affected factor.

## 3 Results

### 3.1 Cohort characteristics

**Figure 1:**
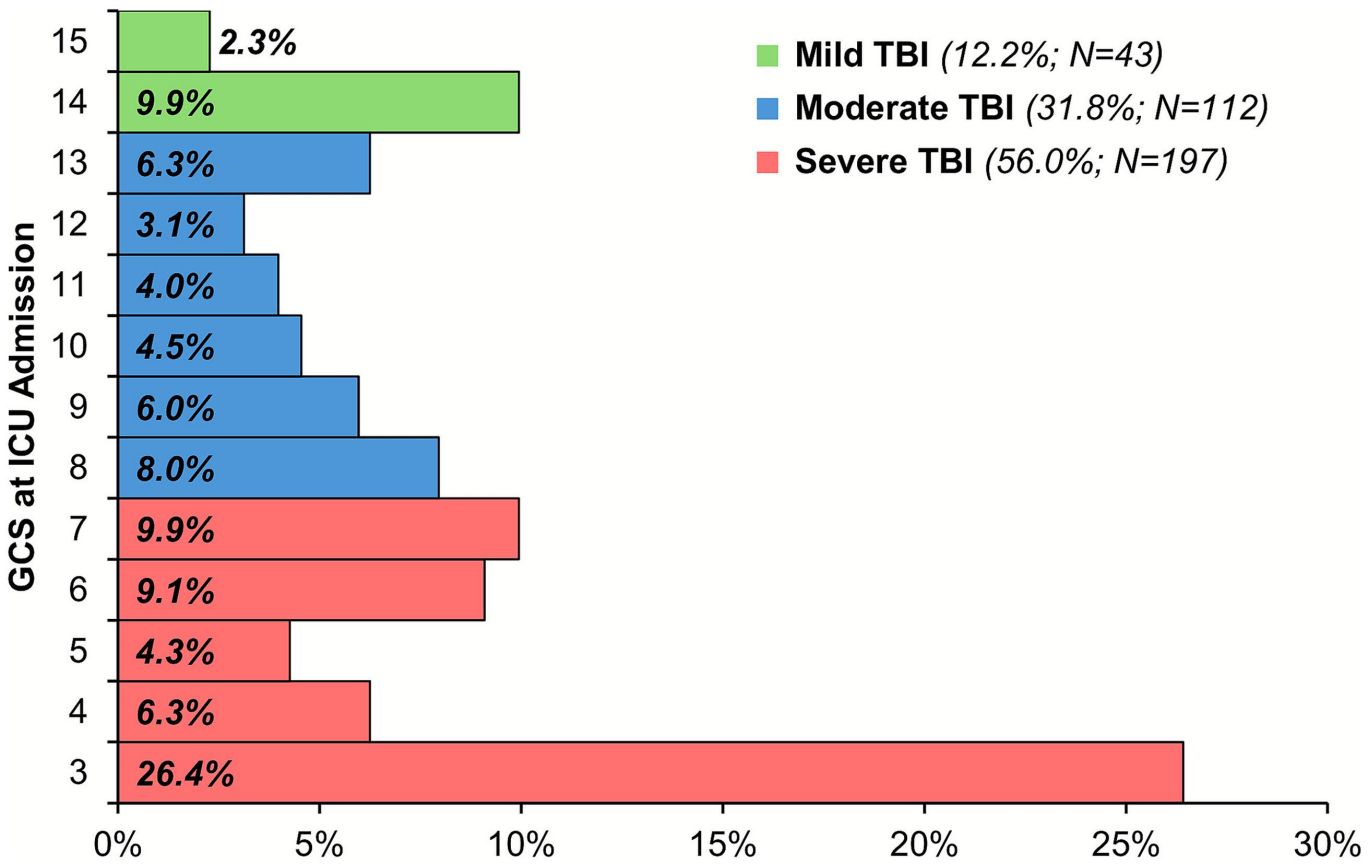
Distribution of GCS at ICU admission. Analysis is based on the 352 patients with a GCS recorded at ICU admission. GCS, Glasgow Coma Scale, ICU, intensive care unit; TBI, traumatic brain injury.

A total of 353 patients were included in the service evaluation, with a mean age at ICU admission of 48 ± 20 years and of whom 77.6% were male; further details of the cohort are reported in Table 1. The GCS at ICU admission was unavailable for one patient; the majority of the remainder (*N* = 352) had severe TBI at ICU admission (56.0%), with 26.4% having a GCS of 3 (Figure 1).

**Table 1:** Cohort characteristics.

Characteristic	Whole cohort (*N* = 353)	TBI severity at ICU admission			*p*-value
		Mild (*N* = 43)	Moderate (*N* = 112)	Severe (*N* = 197)	
Demographics at ICU admission					
Age (Years)	48 ± 20	46 ± 20	49 ± 19	48 ± 20	0.754
Male sex	274 (77.6%)	36 (83.7%)	92 (82.1%)	145 (73.6%)	0.071
APACHE II score	13 ± 6	12 ± 5	13 ± 5	14 ± 6	**0.030**
APACHE II probability (%)	14 (8–26)	11 (8–17)	14 (8–23)	16 (9–31)	**0.002**
Chest injury	119 (33.7%)	11 (25.6%)	30 (26.8%)	78 (39.6%)	**0.015**
Spinal injury	81 (22.9%)	12 (27.9%)	26 (23.2%)	43 (21.8%)	0.459
Lower limb injury	61 (17.3%)	10 (23.3%)	18 (16.1%)	33 (16.8%)	0.693
Pelvic injury	42 (11.9%)	9 (20.9%)	9 (8.0%)	24 (12.2%)	0.760
ICU interventions					
Invasive ventilation	333 (94.6%)	30 (69.8%)	107 (95.5%)	196 (99.5%)	**<0.001**
ICP Bolt	218 (61.8%)	16 (37.2%)	75 (67.0%)	126 (64.0%)	0.160
EVD	27 (7.6%)	1 (2.3%)	7 (6.3%)	19 (9.6%)	0.165
Lumbar drain	33 (9.3%)	1 (2.3%)	10 (8.9%)	21 (10.7%)	0.214
Trache. placed	101 (28.6%)	8 (18.6%)	30 (26.8%)	63 (32.0%)	**0.046**
Days to placement [*N* = 101] ^a^	12 (9–15)	12 (10–15)	12 (9–14)	12 (9–15)	0.746
Trache. removed *[N = 101]* ^b^	77 (76.2%)	6 (75.0%)	25 (83.3%)	46 (73.0%)	0.647
Days to removal [*N* = 77] ^c^	17 (10–24)	19 (17–40)	13 (9–20)	18 (11–28)	0.632

Analyses by TBI severity are based on 352 cases, due to missing data in one patient. Continuous variables are reported as “mean ± standard deviation” or a “median (interquartile range),” as applicable, with *p*-values from Spearman’s rho. Categorical variables are reported as “*N* (%),” with *p*-values from Mann–Whitney U or Kruskal-Wallis tests comparing the GCS at ICU admission between categories (e.g., males vs. females). All *p*-values were calculated using the ungrouped GCS at ICU admission; bold *p*-values are significant at *p* < 0.05. ^a^ Days from ICU admission to tracheostomy placement, in patients with tracheostomies. ^b^ Removal in hospital prior to death or discharge, in patients with tracheostomies. ^c^ Days from placement to removal, in patients where tracheostomies were removed. APACHE II, Acute Physiology and Chronic Health Evaluation II; CSF, cerebrospinal fluid; EVD, extra-ventricular drainage device; ICU, intensive care unit; ICP, intracranial pressure; TBI, traumatic brain injury; Trache., tracheostomy.

### 3.2 Patient outcomes

The ICU mortality rate was 24.9%, with patients surviving to ICU discharge having a median ICU LOS of 13 (IQR: 6–21) days, of which a median of 8 (IQR: 2–15) days were on invasive ventilation (Table 2). A further 4.0% of the cohort died on a hospital ward post-ICU discharge, giving an overall in-hospital mortality rate of 28.9%. Patients surviving to hospital discharge had a median total hospital LOS of 34 (IQR: 20–55) days, with the majority requiring ongoing rehabilitation, either at an inpatient rehabilitation unit (32.3%) or from community rehabilitation services (28.3%). ICU-acquired pneumonia was common, occurring in 43.6% of the cohort.

**Table 2:** Patient outcomes.

Outcome	Whole cohort (*N* = 353)	TBI severity at ICU admission			*p*-value
		Mild (*N* = 43)	Moderate (*N* = 112)	Severe (*N* = 197)	
ICU outcomes					
ICU-acquired pneumonia	154 (43.6%)	16 (37.2%)	52 (46.4%)	86 (43.7%)	0.621
Death in ICU	88 (24.9%)	4 (9.3%)	11 (9.8%)	73 (37.1%)	**<0.001**
ICU LOS (Days) [*N* = 265] ^a^	13 (6–21)	8 (5–16)	12 (6–19)	16 (9–25)	**<0.001**
CCMDS level 3 days [*N* = 265] ^a^	9 (3–16)	3 (0–10)	7 (3–14)	12 (6–17)	**<0.001**
CCMDS level 2 days [*N* = 265] ^a^	3 (1–6)	4 (3–6)	2 (1–5)	3 (1–6)	0.785
Neurological support days[*N* = 265] ^a^	5 (0–8)	1 (0–5)	4 (2–8)	6 (1–10)	**<0.001**
Invasive ventilation days [*N* = 265] ^a^	8 (2–15)	3 (0–10)	7 (2–14)	11 (5–17)	**<0.001**
Sedation days [*N* = 265] ^a^	6 (2–10)	3 (0–7)	4 (2–10)	7 (5–11)	**<0.001**
GCS at ICU discharge [*N* = 209] ^b^					**<0.001** ^**b**^
14–15	141 (67.5%)	29 (85.3%)	64 (73.6%)	47 (54.0%)	
8–13	59 (28.2%)	5 (14.7%)	19 (21.8%)	35 (40.2%)	
3–7	9 (4.3%)	0 (0.0%)	4 (4.6%)	5 (5.7%)	
Hospital outcomes					
Death in hospital	102 (28.9%)	5 (11.6%)	17 (15.2%)	80 (40.6%)	**<0.001**
Hospital LOS (Days) [*N* = 251] ^**c**^	34 (20–55)	30 (15–41)	30 (18–47)	41 (26–64)	**<0.001**
Discharge destination [*N* = 251] ^**c**^					**<0.001**
Inpatient rehabilitation	81 (32.3%)	8 (21.1%)	25 (26.3%)	48 (41.0%)	
Home - community rehabilitation	71 (28.3%)	11 (28.9%)	29 (30.5%)	30 (25.6%)	
Home - no ongoing rehabilitation	35 (13.9%)	10 (26.3%)	18 (18.9%)	7 (6.0%)	
Hospital transfer	64 (25.5%)	9 (23.7%)	23 (24.2%)	32 (27.4%)	

Analyses by TBI severity are based on 352 cases, due to missing data in one patient. Continuous variables are reported as “mean ± standard deviation” or a “median (interquartile range),” as applicable, with *p*-values from Spearman’s rho. Categorical variables are reported as “*N* (%),” with *p*-values from Mann–Whitney U or Kruskal-Wallis tests comparing the GCS at ICU admission between categories (e.g., death: yes vs. no). All *p*-values were calculated using the ungrouped GCS at ICU admission; bold *p*-values are significant at *p* < 0.05. ^a^ In patients surviving to ICU discharge; patients not receiving invasive ventilation or sedation were assigned a value of 0 days for the respective variables. ^b^ Excludes patients who died in ICU, and those that had a tracheostomy at ICU discharge; the *p*-value is from a Spearman’s rho correlation coefficient between the (ungrouped) GCS scores at ICU discharge vs. admission. ^c^ In patients surviving to hospital discharge. CCMDS, Critical Care Minimum Dataset; GCS, Glasgow Coma Scale; ICU, intensive care unit; LOS, length of stay; TBI, traumatic brain injury.

### 3.3 Mobilization data

**Figure 2:**
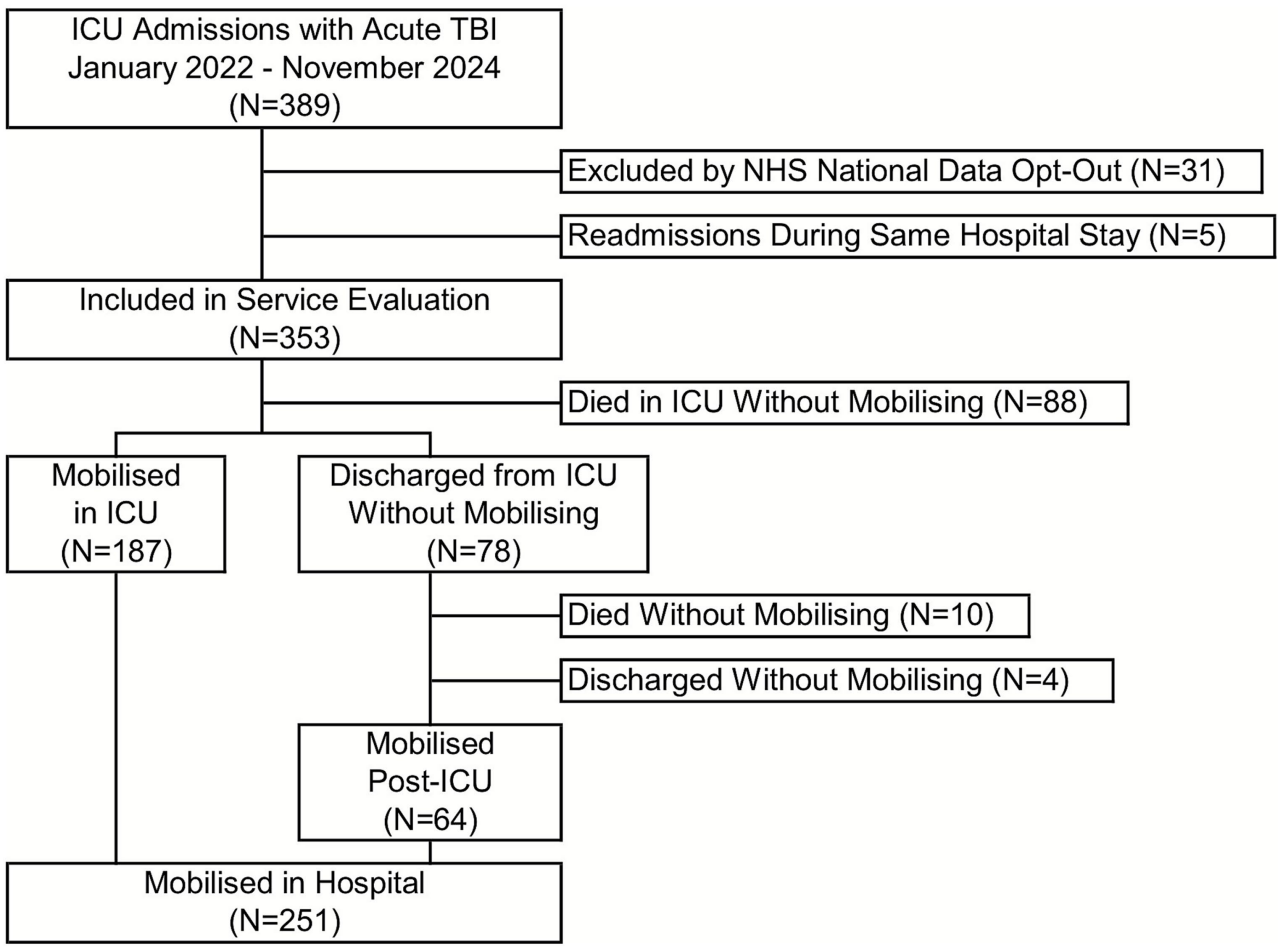
Study flowchart. ICU, intensive care unit, NHS, National Health Service; TBI, traumatic brain injury.

Mobilization was achieved in the ICU for 53.0% of patients, with a further 18.1% first mobilized on a hospital ward post-ICU discharge, giving a total in-hospital mobilization rate of 71.1% (Figure 2). The first mobilization occurred a median of 11 (IQR: 6–18) days after ICU admission, at which time 57.4% had an MMS of 2 (Table 3). When assessed at ICU discharge, the majority of patients had an MMS of 1 or 2 (28.9 and 24.7%, respectively). Only 9.1% of patients achieved an MMS of 7 at ICU discharge, with this increasing to 54.6% at the point of hospital discharge.

**Table 3:** Mobilization outcomes.

Mobilization outcome	Whole cohort (*N* = 353)	TBI severity at ICU admission			*p*-value
		Mild (*N* = 43)	Moderate (*N* = 112)	Severe (*N* = 197)	
Mobilized in hospital	251 (71.1%)	39 (90.7%)	96 (85.7%)	115 (58.4%)	**<0.001** ^**a**^
Days to mobilize [*N* = 251] ^**b**^	11 (6–18)	6 (5–14)	11 (6–17)	13 (8–19)	**<0.001**
MMS at first mobilization [*N* = 251] ^**c**^					**<0.001**
1	-	-	-	-	
2	144 (57.4%)	19 (48.7%)	43 (44.8%)	82 (71.3%)	
3	13 (5.2%)	2 (5.1%)	4 (4.2%)	7 (6.1%)	
4	26 (10.4%)	4 (10.3%)	15 (15.6%)	7 (6.1%)	
5	35 (13.9%)	7 (17.9%)	18 (18.8%)	10 (8.7%)	
6	18 (7.2%)	4 (10.3%)	8 (8.3%)	5 (4.3%)	
7	15 (6.0%)	3 (7.7%)	8 (8.3%)	4 (3.5%)	
MMS at ICU discharge [*N* = 263] ^**d**^					**0.049**
1	76 (28.9%)	6 (15.4%)	37 (36.6%)	33 (27.0%)	
2	65 (24.7%)	10 (25.6%)	21 (20.8%)	34 (27.9%)	
3	33 (12.5%)	4 (10.3%)	6 (5.9%)	23 (18.9%)	
4	22 (8.4%)	4 (10.3%)	5 (5.0%)	13 (10.7%)	
5	30 (11.4%)	6 (15.4%)	17 (16.8%)	7 (5.7%)	
6	13 (4.9%)	3 (7.7%)	7 (6.9%)	2 (1.6%)	
7	24 (9.1%)	6 (15.4%)	8 (7.9%)	10 (8.2%)	
MMS at hospital discharge [*N* = 251] ^**e**^					**<0.001**
1	4 (1.6%)	0 (0.0%)	1 (1.1%)	3 (2.6%)	
2	6 (2.4%)	0 (0.0%)	1 (1.1%)	5 (4.3%)	
3	46 (18.3%)	4 (10.5%)	12 (12.6%)	30 (25.6%)	
4	22 (8.8%)	5 (13.2%)	7 (7.4%)	10 (8.5%)	
5	3 (1.2%)	0 (0.0%)	1 (1.1%)	2 (1.7%)	
6	33 (13.1%)	4 (10.5%)	15 (15.8%)	14 (12.0%)	
7	137 (54.6%)	25 (65.8%)	58 (61.1%)	53 (45.3%)	

Analyses by TBI severity are based on 352 cases, due to missing data in one patient. Data are reported as “*N* (%)” or as “median (interquartile range),” as applicable. *p*-values are from Spearman’s rho, unless stated otherwise, and were calculated using the ungrouped GCS at ICU admission; bold *p*-values are significant at *p* < 0.05. Definitions of each category of the MMS are reported in Figure 5. ^a^ *p*-value from Mann–Whitney U test comparing the GCS at ICU admission between patients that did vs. did not achieve mobilization. ^b^ Time from ICU admission to mobilization, in patients who mobilized in hospital. ^c^ In patients who mobilized in hospital. ^d^ In patients surviving to ICU discharge; additionally excludes two patients for whom data were not recorded. ^e^ In patients surviving to hospital discharge. ICU, intensive care unit; MMS, Manchester Mobility Scale; TBI, traumatic brain injury.

### 3.4 Associations with TBI severity

**Figure 3:**
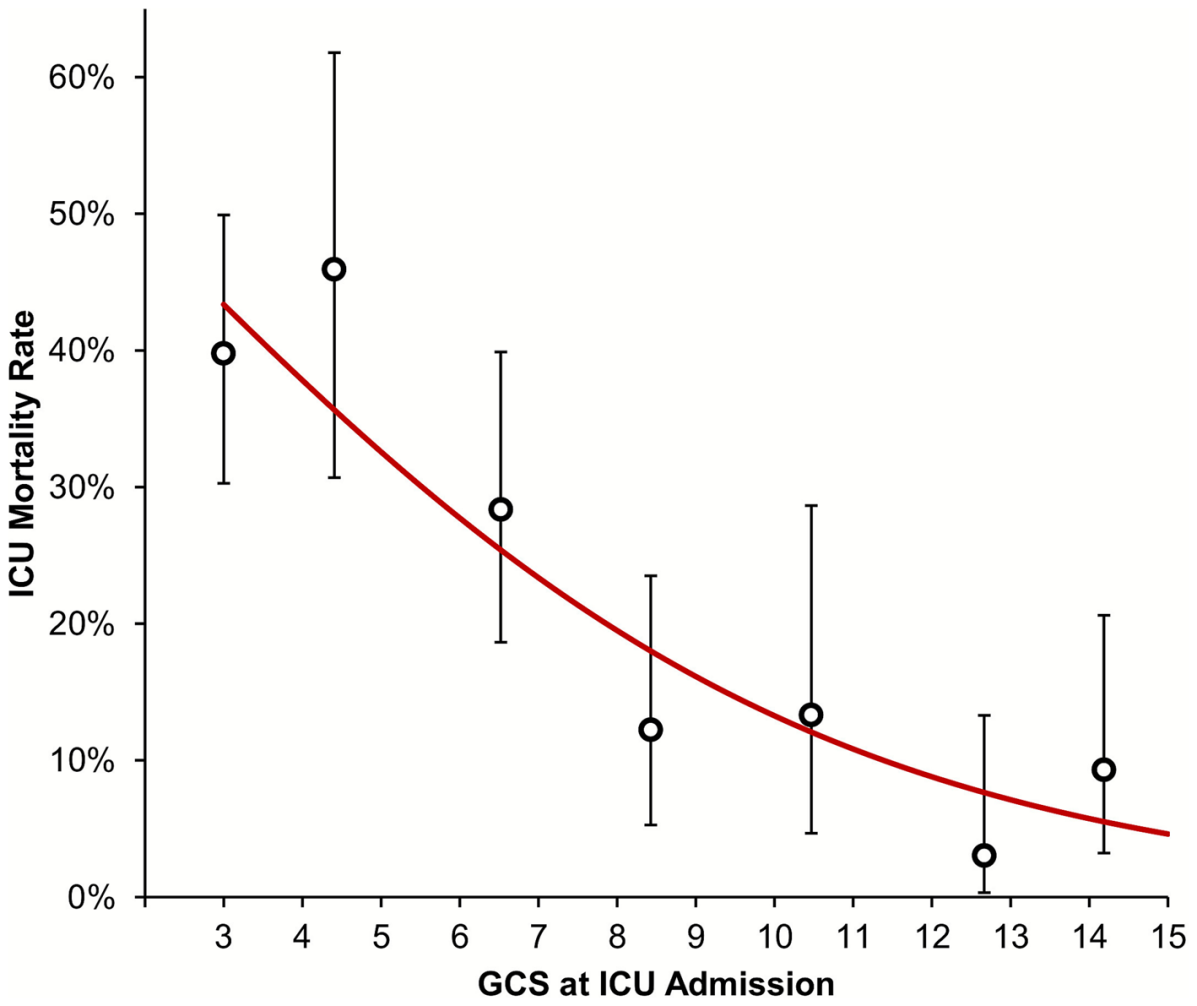
Association between GCS at ICU admission and ICU mortality. The trend line is from a binary logistic regression model, with the GCS as a continuous covariate. To visualize the goodness of fit of the model, points are additionally plotted representing the observed ICU mortality rates within subgroups of GCS (namely: 3, 4–5, 6–7, 8–9, 10–11, 12–13, and 14–15), with whiskers representing 95% confidence intervals. GCS, Glasgow Coma Scale; ICU, intensive care unit.

Neither patient age (*p* = 0.754) nor sex (*p* = 0.071) were found to differ significantly by TBI severity (Table 1). However, APACHE II scores (*p* = 0.030) and rates of chest injury (*p* = 0.015) both increased progressively with TBI severity. Increasing TBI severity was associated with a higher rate of invasive ventilation (*p* < 0.001) and tracheostomy placement (*p* = 0.046). However, no significant associations with either the timing of tracheostomy placement (*p* = 0.746); rate of tracheostomy removal (*p* = 0.647); or timing of the removal (*p* = 0.632) were observed. The ICU mortality rate increased significantly with TBI severity (*p* < 0.001; Figure 3), from 9.3% in those with mild TBI to 37.1% in severe TBI. In patients surviving ICU, the ICU LOS increased significantly with TBI severity (*p* < 0.001), with medians of 8, 12 and 16 days for mild, moderate and severe TBIs, respectively; corresponding increases in the numbers of CCMDS Level 3 days, and durations of neurological support, invasive ventilation and sedation in ICU were also observed (*p* < 0.001 for all variables; Table 2).

**Figure 4:**
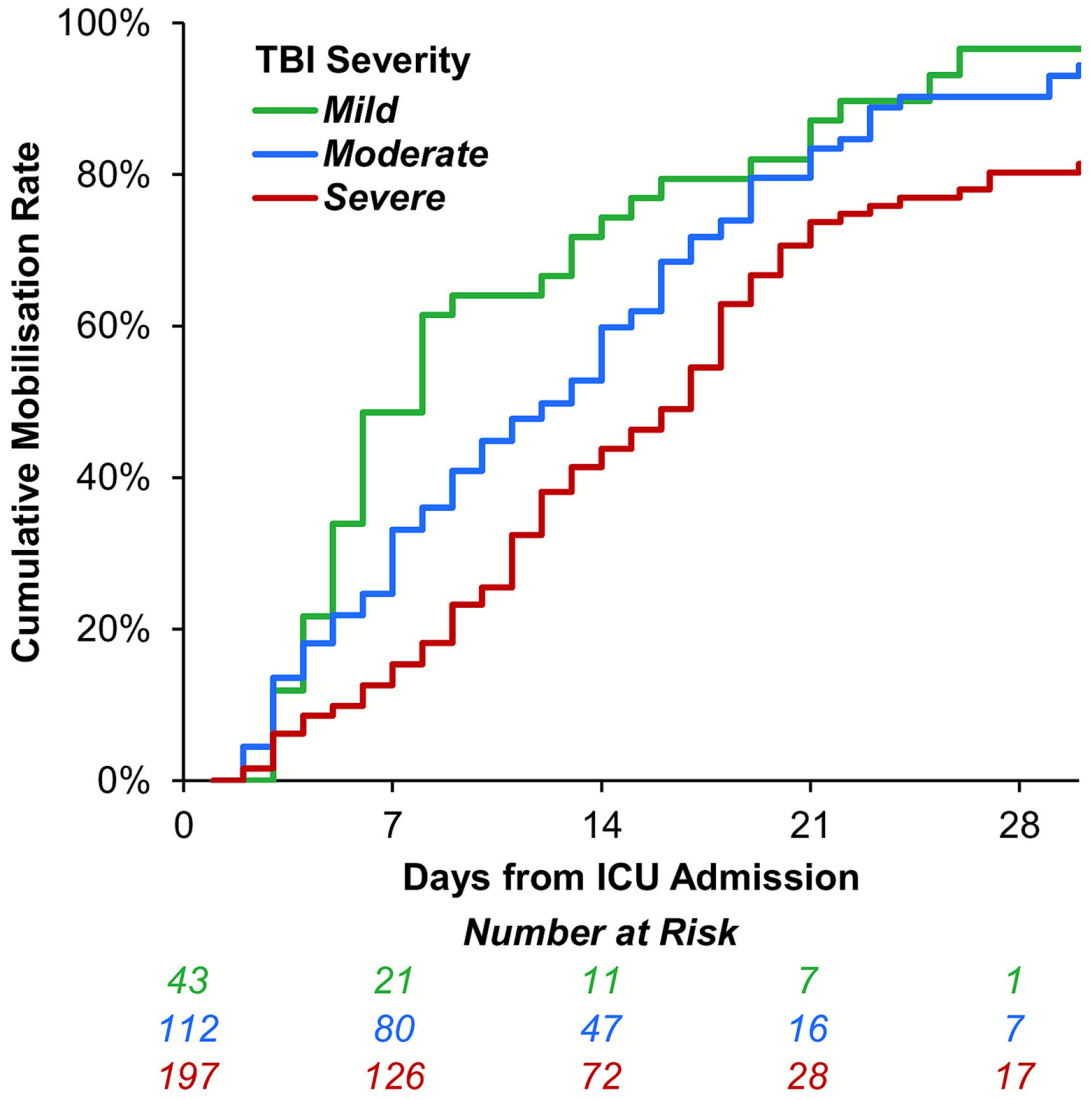
Kaplan–Meier curve of time to mobilization by TBI severity at ICU admission. Data were analyzed using a time-to-event approach, with mobilization as the event of interest; follow-up commenced at ICU admission, and patients were censored at death or hospital discharge. The x-axis was truncated at 30 days, due to declining numbers at risk. ICU, intensive care unit; TBI, traumatic brain injury.

**Figure 5:**
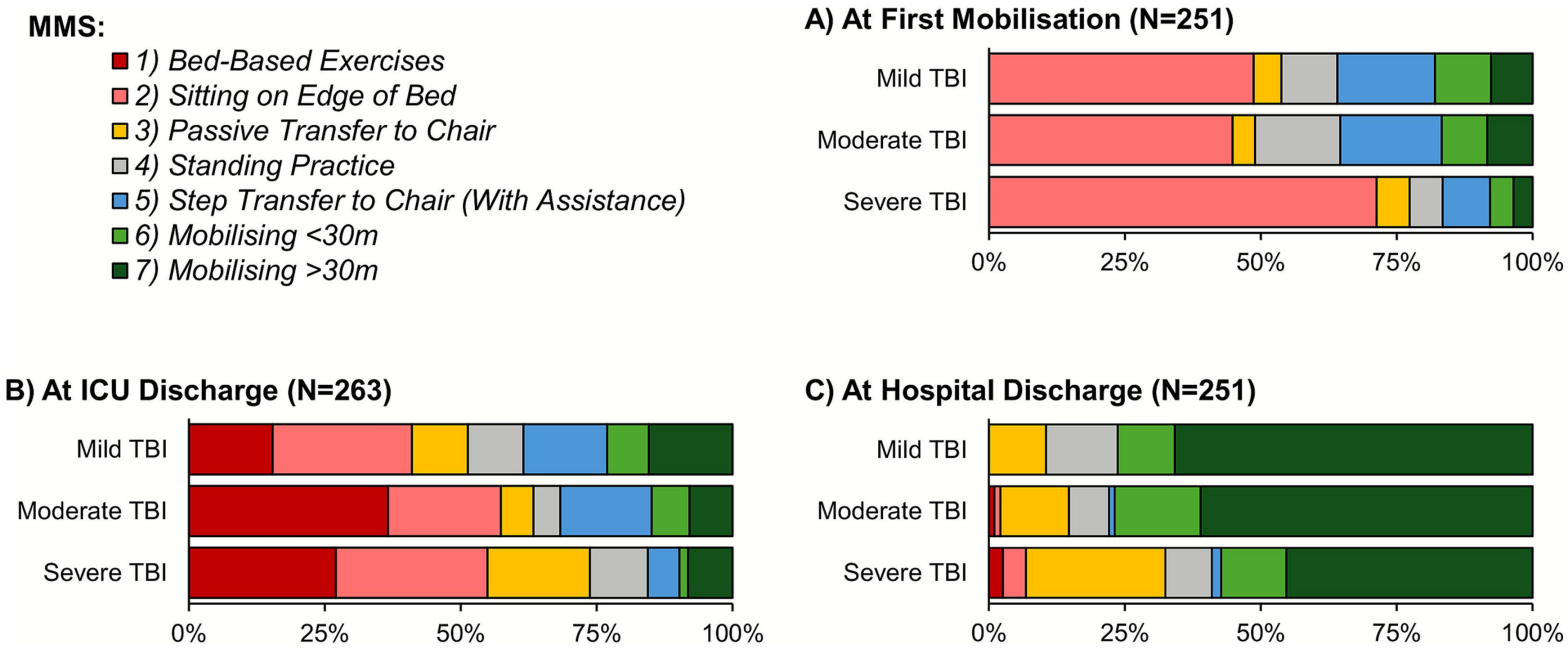
MMS by TBI severity. Analyses are based on the subgroups of patients who were mobilized, discharged from ICU, or discharged from hospital for **(A–C)**, respectively; see Table 3 for further details. ICU, intensive care unit; MMS, Manchester Mobility Scale; TBI, traumatic brain injury.

In-hospital mobilization rates declined significantly with TBI severity (90.7% vs. 58.4% for mild vs. severe TBI; *p* < 0.001; Table 3). In patients where mobilization was achieved, the time to the first mobilization increased significantly with TBI severity (median: 6 vs. 13 days for mild vs. severe TBI; *p* < 0.001). Mobilization was also assessed using a Kaplan–Meier approach, to account for the incomplete follow-up of patients who died or were transferred prior to mobilizing. This returned consistent results, with estimated mobilization rates within 14 days of ICU admission of 74.3, 59.8 and 43.8% for mild, moderate and severe TBI, respectively (Figure 4). The MMS at the first mobilization decreased significantly with the TBI severity (*p* < 0.001; Figure 5A), with similar trends observed for the MMS at both ICU and hospital discharge (Figures 5B,C).

### 3.5 Associations with post-ICU mobilization

Of the 251 patients who were mobilized in hospital, the first mobilization occurred in ICU in 74.5%, with the remaining 25.5% mobilized at a median of 4 days (IQR: 3–9) after ICU discharge (i.e., “post-ICU”; Figure 2; Table 4). Those mobilized after ICU discharge were mobilized significantly later than those mobilized on ICU, at a median of 16 vs. 11 days after admission (*p* < 0.001, Table 5); however, these patients had a significantly higher MMS at this time (MMS > 2 in 60.9% vs. 36.4%, *p* = 0.009).

**Table 4:** Demographics and ICU treatment by timing of first mobilization.

Characteristic	Timing of first mobilization		*p*-value
	In ICU (*N* = 187)	Post-ICU (*N* = 64)	
Demographics at ICU admission			
Age (Years)	45 ± 18	43 ± 17	0.361
Male sex	153 (81.8%)	52 (81.3%)	1.000
APACHE II score	12 ± 5	11 ± 5	**0.039**
APACHE II probability (%)	13 (8–22)	11 (7–18)	0.144
TBI severity [*N* = 250] ^a^			0.837 ^b^
Mild	33 (17.7%)	6 (9.4%)	
Moderate	64 (34.4%)	32 (50.0%)	
Severe	89 (47.8%)	26 (40.6%)	
Chest injury	51 (27.3%)	24 (37.5%)	0.154
Spinal injury	32 (17.1%)	24 (37.5%)	**0.001**
Lower limb injury	26 (13.9%)	16 (25.0%)	0.052
Pelvic *I*njury	14 (7.5%)	10 (15.6%)	0.082
ICU interventions			
Invasive ventilation	175 (93.6%)	58 (90.6%)	0.411
ICP Bolt	124 (66.3%)	41 (64.1%)	0.762
EVD	12 (6.4%)	7 (10.9%)	0.274
Lumbar drain	20 (10.7%)	7 (10.9%)	1.000
Tracheostomy placed	80 (42.8%)	14 (21.9%)	**0.003**

Continuous variables are reported as “mean ± standard deviation” or a “median (interquartile range),” as applicable, with *p*-values from Mann–Whitney U tests. Categorical variables are reported as “*N* (%),” with *p*-values from Fisher’s exact tests. Bold *p*-values are significant at *p* < 0.05. ^a^ Based on 250 patients, due to missing data. ^b^ *p*-value from Mann–Whitney U test, as the factor is ordinal. APACHE II, Acute Physiology and Chronic Health Evaluation II; CSF, cerebrospinal fluid; EVD, extra-ventricular drainage device; ICU, intensive care unit; ICP, intracranial pressure; TBI, traumatic brain injury.

**Table 5:** Outcomes by timing of first mobilization.

Outcome	Timing of first mobilization		*p*-value
	In ICU (*N* = 187)	Post-ICU (*N* = 64)	
ICU-acquired pneumonia	101 (54.0%)	28 (43.8%)	0.192
ICU LOS (Days)	14 (7–24)	12 (6–16)	**0.014**
Hospital LOS (Days)	34 (19–54)	36 (26–65)	0.172
CCMDS level 3 days	9 (3–17)	9 (3–15)	0.336
CCMDS level 2 days	4 (2–7)	2 (1–4)	**<0.001**
Neurological support days	5 (1–8)	4 (0–9)	0.618
Invasive ventilation days	8 (2–16)	7 (3–13)	0.269
Sedation days	6 (2–10)	5 (3–10)	0.658
GCS at ICU discharge [*N* = 196] ^a^			**<0.001** ^**a**^
14–15	108 (75.5%)	31 (58.5%)	
8–13	35 (24.5%)	20 (37.7%)	
3–7	0 (0.0%)	2 (3.8%)	
Weekday of ICU discharge			**0.013**
Monday	34 (18.2%)	4 (6.3%)	
Tuesday	25 (13.4%)	9 (14.1%)	
Wednesday	28 (15.0%)	10 (15.6%)	
Thursday	33 (17.6%)	5 (7.8%)	
Friday	30 (16.0%)	12 (18.8%)	
Saturday	26 (13.9%)	13 (20.3%)	
Sunday	11 (5.9%)	11 (17.2%)	
Time to first mobilize (Days)	11 (5–17)	16 (8–28)	**<0.001**
MMS at first mobilization			**0.009** ^**b**^
2	119 (63.6%)	25 (39.1%)	
3	2 (1.1%)	11 (17.2%)	
4	17 (9.1%)	9 (14.1%)	
5	30 (16.0%)	5 (7.8%)	
6	7 (3.7%)	11 (17.2%)	
7	12 (6.4%)	3 (4.7%)	
MMS at hospital discharge [*N* = 247] ^c^			0.153^b^
2	4 (2.2%)	2 (3.1%)	
3	32 (17.5%)	14 (21.9%)	
4	16 (8.7%)	6 (9.4%)	
5	3 (1.6%)	0 (0.0%)	
6	21 (11.5%)	12 (18.8%)	
7	107 (58.5%)	30 (46.9%)	

Continuous variables are reported as “mean ± standard deviation” or a “median (interquartile range),” as applicable, with *p*-values from Mann–Whitney U tests. Categorical variables are reported as “*N* (%),” with *p*-values from Fisher’s exact tests. Bold *p*-values are significant at *p* < 0.05. Definitions of each category of the MMS are reported in Figure 5. ^a^ Excludes patients who had a tracheostomy at ICU discharge; the *p*-value is from a Mann–Whitney U test on the (ungrouped) GCS scores. ^b^ *p*-value from Mann–Whitney U test, as the factor is ordinal. ^c^ Based on 247 patients, due to missing data. CCMDS, Critical Care Minimum Dataset; GCS, Glasgow Coma Scale; ICU, intensive care unit; LOS, length of stay; MMS, Manchester Mobility Scale; TBI, traumatic brain injury.

Comparisons between the two subgroups found that patients who mobilized post-ICU had significantly lower APACHE II scores at admission (*p* = 0.039); were more likely to have a spinal injury (*p* = 0.001); and less likely to have had a tracheostomy placed (*p* = 0.003; Table 4). For the subgroup of patients without tracheostomies at ICU discharge, the GCS at this time was significantly lower in patients who were mobilized post-ICU (*p* < 0.001). Patients mobilized post-ICU had a significantly shorter ICU LOS (median: 12 vs. 14 days, *p* = 0.014) and fewer CCMDS Level 2 days (median: 2 vs. 4 days, *p* < 0.001). A significant difference in the weekday of ICU discharge was also observed (*p* = 0.013), with post-ICU mobilization more likely in patients discharged from ICU on a Sunday (50%; 11/22) and less likely in those discharged on a Monday (11%; 4/38; Table 5).

A case note review was then performed for the 64 patients mobilized post-ICU, which identified staffing limitations to be the most common reason for mobilization not being achieved in ICU (31%; *N* = 20), followed by awaiting an injury management plan (20%; *N* = 13). The remaining cases were not deemed appropriate for mobilization prior to ICU discharge, either due to injury restrictions that prevented out-of-bed mobilization (20%; *N* = 13), neurological reasons (14%; *N* = 9), medical reasons (9%; *N* = 6), or the presence of an EVD (5%; *N* = 3).

## 4 Discussion

The aim of this service evaluation was to benchmark the current mobilization activity of TBI patients admitted to the ICU at a UK Level 1 Trauma Center. To our knowledge, this is the first report of mobilization activity in the neurotrauma cohort from the UK, with limited existing literature exploring out-of-bed mobilization in the TBI population worldwide. Our results identified that approximately half of all TBI patients were not mobilized while in the ICU. Among those who were mobilized, the first mobilization occurred at a median of 11 days after ICU admission, despite sedation and mechanical ventilation ceasing at a median of 6 days and 8 days, respectively. At ICU discharge, mobility status was low among TBI patients, with the majority scoring an MMS of 1 or 2 (28.9 and 24.7%, respectively) and only 9.1% of patients achieving an MMS of 7. TBI severity significantly impacted the mobilization rates and mobility status achieved at both ICU and hospital discharge. Outcomes for TBI patients were generally poor, with almost a third of patients not surviving to hospital discharge, while survivors faced an average of 34 days in hospital, with the majority requiring ongoing rehabilitation. The results of our service evaluation highlight an urgent need for further prospective studies of rehabilitation specific to the neurotrauma population, to improve mobilization practices and outcomes for TBI patients admitted to the ICU.

### 4.1 Mobilization rates

Mobilization in the ICU was achieved in 53.0% of patients. As such, almost half of all TBI patients admitted to the ICU over a three-year evaluation period were not mobilized prior to ICU discharge. The low levels of in-ICU mobilization activity may be reflective of a critically unwell and acutely injured cohort, whereby rehabilitation is not deemed safe nor appropriate, with 24.9% of patients not surviving their ICU admission. Existing safety criteria advise against out-of-bed mobilization when pertinent factors relevant to the neurotrauma cohort are present, such as ICP monitoring, spinal precautions, unstable pelvic injuries or long-bone fractures (52, 53). In our cohort, 22.9% of TBI patients also sustained a spinal injury, 17.3% sustained lower limb fractures and 11.9% sustained a pelvic injury, potentially explaining the limited out-of-bed mobilization due to these injury restrictions. In addition, those who were first mobilized post-ICU discharge were significantly more likely to have presented with a spinal injury, further suggestive that bed-rest precautions impeded in-ICU mobilization. Likewise, over 60% of the cohort required an ICP bolt; suggesting a period of acute neurological instability, when the patient requires close surveillance of neurological and haemodynamic status (54, 55). Interestingly, the average period of neurological support was 5 days, suggesting that ICP monitoring may only be a barrier to rehabilitation during a transient and hyper-acute period post-injury. Still, the safety of early mobilization in the neurotrauma ICU may also be impeded by other potential complications during the acute recovery period after TBI, such as seizures, intracranial hypertension, haematoma expansion, paroxysmal sympathetic hyperactivity or cerebral perfusion management (10).

Of those who were mobilized in hospital, this occurred post-ICU discharge in 25.5%, by a median of 4 days after transfer from ICU. Interestingly, this group included patients with a higher incidence of ICU discharge on a Sunday, potentially coinciding with limited therapy provision of rehabilitation over a weekend and lack of seven-day rehabilitation service. Case note review of the post-ICU mobilization group identified staffing limitations as the primary documented reason for mobilization not taking place in the ICU. However, the clinical reasoning and therapist decision making was not interrogated as part of this retrospective service evaluation and, therefore, causality for the low ICU mobilization rates is speculative. Nonetheless, with known benefits of ICU mobilization upon patient functional outcomes, length of ICU admission and subsequent healthcare costs, further prospective research must be performed to explore the limitations and barriers to out-of-bed mobilization in the neurotrauma ICU.

While this study was primarily concerned with benchmarking current mobilization activity, there are many challenges to early mobilization, which have been identified by others (10). For example, patients may exhibit altered consciousness, impaired cognition, motor dysfunction, haemodynamic instability, and susceptibility to secondary neurological injuries, requiring caution to minimize adverse effects (56, 57). Other factors such as delirium, risk of falls, activity restrictions due to surgical interventions such as invasive drains and lines as well as staffing and resources have all been recognized as factors that affect early mobilization protocols (58–60). Despite such challenges, with most patients in our cohort scoring MMS 1 or 2 at ICU discharge, there remains scope for improvement in those limited to passive mobilization techniques, enabling passive transfer to a chair and thus scoring MMS 3. However, mobilization practices are not standardized, and further research is needed to determine the optimal timing and dosage of mobility to improve long-term outcomes in neurotrauma ICU patients.

### 4.2 Time to mobilize in the neurotrauma ICU

Among those who did mobilize, the first mobilization occurred a median of 11 days after ICU admission. This time to mobilize is similar to two previous studies from other neurotrauma ICUs, which assessed the safety and feasibility of commencing mobilization 12 days after brain injury using a tilt-table verticalization stepping-device (25, 33). However, a previous study of early ICU rehabilitation from our institution identified a median time to mobilize for a general ICU population of 8 days in those receiving enhanced rehabilitation, despite having higher APACHE-II scores and receiving longer periods of sedation and mechanical ventilation than the current TBI cohort (13). Interestingly, sedation and mechanical ventilation ceased at a median of 6 days and 8 days, respectively, in this TBI cohort, suggesting a potential for rehabilitation to commence earlier than day 11; yet further exploration of sedation practices and barriers to mobilization are required. In addition, almost a third of all patients had a tracheostomy placed, which may have enabled sedation weaning and earlier mobilization, despite the ongoing need for airway and ventilatory support. Mobilization is safe and feasible while patients are still mechanically ventilated, with previous studies from the medical ICU commencing rehabilitation within 36 h of intubation (61–63). ICU guidelines (including “A to F bundles”) recommend a coordinated multidisciplinary approach to awakening from sedation, spontaneous breathing trials and mobilization for enhanced patient outcomes (64–67). However, there has been limited research to explore the utility of such bundles in the neurotrauma ICU, perhaps limiting their application into practice with TBI populations (68). The lack of rehabilitation protocols specific to the neurotrauma ICU population means that the timing of mobilization after TBI remains controversial (25, 33). Therefore, larger, high-quality studies are warranted to determine the optimal timing of mobilization after TBI.

TBI severity was significantly associated with the time to mobilization, in addition to in-hospital mobilization rates and the mobility status achieved at both ICU and hospital discharge. Severe TBI may limit rehabilitation due to impaired cerebral autoregulation, associated with haemodynamic instability, elevated ICP and cerebral ischaemia (10, 39). If cerebral haemodynamic regulatory mechanisms are insufficient to maintain cerebral perfusion, changes in posture through mobilization may jeopardize cerebral oxygen delivery and, thus, potentially cause harm (69). Although postural changes are known to influence ICP, previous studies have suggested that upright positioning, once the patient has stabilized following acute injury, improves venous outflow without adverse effects upon ICP (20, 24). Additionally, small studies of orthostatic exercise after TBI have shown this to be safe, feasible and effective (39). Likewise, immobilization after injury may exacerbate haemodynamic instability and lead to the development of orthostatic hypotension (39). However, the recent AVERT study demonstrated less favorable outcomes in the intervention group of an early rehabilitation trial after acute stroke; potentially since patients were mobilized out of bed within 24 h of insult while still in the acute and, likely unstable, phase of brain injury (70). Despite such controversies, a recent clinician survey of ICU mobilization practices demonstrated unanimous favor for mobilization of TBI patients in the ICU (71). While opinions differed on the optimal timing for commencing rehabilitation, these were often based on patient haemodynamic stability (71). Although ICU guidelines recommend ‘early’ rehabilitation in the ICU, the specific timing may not be quantifiable, due to vast, heterogenous patient- and therapy-related factors in the ICU (8). On balance, the complex and unique circumstances of the TBI cohort mean that mobilization should be carefully and cautiously considered an individual case-by-case basis, once the patient has haemodynamically stabilized after acute injury and following comprehensive risk assessment.

### 4.3 Mobility status at ICU discharge

Mobility levels among TBI patients were low, with the majority of patients scoring an MMS of 1 or 2 (28.9 and 24.7%, respectively) at ICU discharge. In previous rehabilitation studies from our institution, general ICU patients had an average MMS score of 5 at ICU discharge, with 47% achieving an MMS of 6–7, which increased to 73% with the introduction of early, structured rehabilitation (13). However, in our TBI cohort, only 14.1% of patients achieved an MMS of 6–7 at the point of ICU discharge. While TBI patients appear to be discharged from the ICU with lower mobility levels than the general ICU cohort, the effects of TBI on cognitive and motor functions present a number of unique challenges to mobilization (10). Although many ICU patients face profound weakness due to deleterious effects of critical illness on muscle mass and function, neurotrauma patients may face specific neuromuscular and sensory issues that cause postural and gait disturbances, such as hemiparesis, ataxia or spasticity (72, 73). Specialist seating may therefore be required in order to progress mobility status beyond as MMS of 2. Cognitive changes, such as post-traumatic amnesia, dyspraxia or aphasia, alongside fatigue and sleep disturbances may further impair the TBI patient’s ability to engage in rehabilitation or safely mobilize. However, in our cohort, over two thirds had a GCS score of 14 to 15 by the time of ICU discharge, suggesting their potential for engagement in rehabilitation. Nonetheless, structured, multidisciplinary ICU rehabilitation of the TBI patient should consider wider components of cognitive and functional recovery, beyond mobilization alone (28). This therefore questions the relevancy of the MMS scale to the neurotrauma population, although it is a validated outcome measure of ICU physical function (51).

Previous studies of neurotrauma rehabilitation have used a vast array of functional outcome measures, including the Disability Rating Scale, Levels of Cognitive Functioning and Wessex Head Injury Matrix (33, 39, 74). Other measures may better quantify the rehabilitation outcomes of ICU TBI patients, yet currently the most appropriate functional outcome for this cohort is unknown. Nonetheless, it appears that TBI patients have longer periods of immobilization than general ICU patients. The adverse consequences of immobilization have been widely documented, including neuromuscular weakness and muscle atrophy, pressure sores and VTE, with increased risk of hospital acquired infections such as pneumonia, and prolonged hospital stays (28, 75). Mobilization status is of pertinent interest due its close association with ICU patient outcomes, including duration of mechanical ventilation and length of ICU admission (11–14, 62, 76, 77). With the average cost of an ICU bed in the UK’s NHS equating to more than £1,500 per night, interventions to reduce the ICU care needs of TBI patients is of significant importance in modern healthcare (78).

### 4.4 Outcomes for TBI patients admitted to the ICU

In keeping with previous literature from the neurotrauma population, outcomes for TBI patients admitted to the ICU were generally poor (31, 79, 80). During our service evaluation period, over a quarter of patients did not survive to hospital discharge. Survivors faced a median of 34 days in hospital, with the majority requiring ongoing rehabilitation beyond discharge. There are numerous heterogenous factors that may influence patient outcomes after neurotrauma, including pre-hospital care and in-hospital management including type of ICU admission, pre-morbid health status, glycaemic control and injury severity (5, 81). Interestingly, despite a high volume of TBI patients being admitted to our ICU, previous studies have found no association between annual TBI volume and ICU mortality (82).

However, previous studies have found increased mobility levels (defined as an MMS ≥ 5) at ICU discharge to be associated with reduced hospital length of stay and discharge to usual residence (83). In-ICU rehabilitation studies in the neurotrauma population have reported improvements in consciousness and disability at ICU discharge with the use of tilt-table stepping devices and mobilization programs (25, 33, 84, 85). However, recent systematic reviews of mobilization in patients following traumatic injury, and also in those with severe acquired brain injury (not exclusive to TBI), found inconsistent results, possibly due to heterogeneity in patient populations, rehabilitation interventions and outcome measures (29, 86).

### 4.5 Limitations

This service evaluation had several strengths, including the large sample size and near-complete data for all variables of interest. However, there are also some limitations which need to be considered when interpreting the findings. Primarily, the single-center service evaluation design meant that the results are only directly applicable to our institution. Consequently, the findings may not be generalisable to other centers, particularly those with very different case-mixes, or using different rehabilitation protocols. Secondly, analysis of mobility outcomes only included patients who survived to discharge. Given the relatively high mortality rate in patients presenting to ICU with TBIs (28.9% in the present cohort), excluding these patients is likely to have introduced selection bias. As such, while the findings of these analyses will be applicable to the subgroup of patients that survived to discharge, they cannot be reliably generalized to the broader cohort of patients presenting to ICU with TBIs. Similarly, of the subgroup of patients surviving to hospital discharge, 25.5% were transferred to a different hospital for ongoing inpatient treatment; hence, would have an incomplete period of follow-up when assessing outcomes. However, since only four of these patients had failed to mobilize prior to transferring, it is likely that any bias would have negligible impact on the analyses of functional outcomes.

## 5 Conclusion

This service evaluation is the first known report of current mobilization activity specific to TBI patients admitted to the ICU. Our results identified that approximately half of all TBI patients at our institution did not mobilize while in the ICU, achieving low mobility status at ICU discharge. Outcomes for this cohort of TBI patients were generally poor, with the majority requiring ongoing rehabilitation beyond hospital discharge. The results of our service evaluation highlight an urgent need for prospective rehabilitation studies specific to the neurotrauma population; identifying and addressing the barriers to early mobilization and evaluating the safety and feasibility of rehabilitation interventions for TBI patients admitted to the ICU. Specific neurotrauma guidance to support safe and appropriate decision-making for ‘earlier’ ICU rehabilitation is urgently required, which promotes an ethos of early mobilization, while emphasizing appropriate safety measures, monitoring and therapeutic benefit. This could be the first step toward developing a national consensus statement for early mobilization in the neurocritical care for acute TBI patients. However, future guidelines need further large-scale studies to support definitive recommendations.

## Data Availability

The data analyzed in this study is subject to the following licenses/restrictions: de-identified data from this study are not available in a public archive. De-identified data from this study will be considered to be made available by emailing the corresponding author. Requests to access these datasets should be directed to fjh722@student.bham.ac.uk; jonathan.weblin@uhb.nhs.uk.

## Glossary

APACHE: Acute Physiology and Chronic Health Evaluation
CCMDS: critical care minimum dataset
CSF: cerebrospinal fluid
CT: computed tomography
EHR: electronic health records system
EVD: extra-ventricular drainage device
GCS: Glasgow Coma Scale;
HAP: hospital-acquired pneumonia;
ICNARC: Intensive Care National Audit and Research Center
ICP: intracranial pressure monitoring
ICU: intensive care unit
IQR: interquartile range
LOS: length of stay
MMS: Manchester Mobility Scale
NHS: National Health Service
QEHB: Queen Elizabeth Hospital Birmingham
SD: standard deviation
TBI: traumatic brain injury
UK: United Kingdom
VAP: ventilator-associated pneumonia
